# Influenza surveillance: determining the epidemic threshold for influenza by using the Moving Epidemic Method (MEM), Montenegro, 2010/11 to 2017/18 influenza seasons

**DOI:** 10.2807/1560-7917.ES.2019.24.12.1800042

**Published:** 2019-03-21

**Authors:** Bozidarka Rakocevic, Anita Grgurevic, Goran Trajkovic, Boban Mugosa, Sandra Sipetic Grujicic, Sanja Medenica, Olivera Bojovic, José Eugenio Lozano Alonso, Tomas Vega

**Affiliations:** 1 Center for Disease Control and Prevention, Institute of Public Health, Podgorica, Montenegro; 2 These authors contributed equally to this work; 3 Institute of Epidemiology, Faculty of Medicine, University of Belgrade, Belgrade, Serbia; 4 Institute for Medical Statistics and Informatics, Faculty of Medicine, University of Belgrade, Belgrade, Serbia; 5 Department for Tuberculosis, Hospital for Lung Disease and Tuberculosis Brezovik, Niksic, Montenegro; 6 Public Health Directorate, Castilla y León Regional Health Ministry, Valladolid, Spain

**Keywords:** Influenza-like illnesses, threshold, incidence, surveillance, Montenegro

## Abstract

Background: In 2009, an improved influenza surveillance system was implemented and weekly reporting to the World Health Organization on influenza-like illness (ILI) began. The goals of the surveillance system are to monitor and analyse the intensity of influenza activity, to provide timely information about circulating strains and to help in establishing preventive and control measures. In addition, the system is useful for comparative analysis of influenza data from Montenegro with other countries.

Aim: We aimed to evaluate the performance and usefulness of the Moving Epidemic Method (MEM), for use in the influenza surveillance system in Montenegro.

Methods: Historical ILI data from 2010/11 to 2017/18 influenza seasons were modelled with MEM. Epidemic threshold for Montenegro 2017/18 season was calculated using incidence rates from 2010/11–2016/17 influenza seasons.

Results: Pre-epidemic ILI threshold per 100,000 population was 19.23, while the post-epidemic threshold was 17.55. Using MEM, we identified an epidemic of 10 weeks’ duration. The sensitivity of the MEM epidemic threshold in Montenegro was 89% and the warning signal specificity was 99%.

Conclusions: Our study marks the first attempt to determine the pre/post-epidemic threshold values for the epidemic period in Montenegro. The findings will allow a more detailed examination of the influenza-related epidemiological situation, timely detection of epidemic and contribute to the development of more efficient measures for disease prevention and control aimed at reducing the influenza-associated morbidity and mortality.

## Introduction

Influenza is a highly infectious viral disease that represents a considerable public health problem, as it is a cause of significant morbidity and mortality rates globally [1,2]; the most severe clinical symptoms and mortality occur in high-risk populations [3]. Influenza also places an important socioeconomic burden on society, owing to the high cost of treatment and a decrease in work productivity when an individual is infected [4,5]. During the influenza season, factors such as individual awareness of the disease, social behaviour, climate, spread of influenza virus infection and discrepancies between the vaccine strain and the circulating strain of influenza virus (leading to decreased vaccine effectiveness) etc. act in concert to create an influence epidemic [6]. The severity and duration of the influenza epidemic vary every year due to differences in virus circulation, population susceptibility and climatic factors; in addition, the onset, duration, intensity, geographic spread of influenza and the severity of the disease are often unpredictable [7-10].

The specific goal of influenza surveillance is to provide timely and high-quality epidemiological data to reduce the impact of illness and to inform public health authorities in their appropriate response to this disease. Two important benefits include the comparison of data from the current influenza season to previous seasons and the identification of an increased activity during a specific time frame, which could represent the onset of an influenza epidemic. A specific target of influenza control is the identification of epidemic thresholds that will determine the start and end of an epidemic [11]. In order to support public health authorities in anticipating onset of influenza epidemics and initiating an appropriate and timely response, the concept of epidemic thresholds is applied in a large number of national monitoring programs [12]. There are numerous methods for calculating the onset of an influenza epidemic in the season using different data sources [9,13]. According to the World Epidemiological Standards for the Influenza Surveillance, the World Health Organization (WHO) provides several methods for determining the epidemic threshold, one of which is visual, process control and averaging [11]; Moving Epidemic Method (MEM) is an example of averaging method. For example, in Spain, a model for detection of seasonal epidemics has been applied since 2003 and a modified version of MEM has been implemented by the European Centre for Disease Control and Prevention (ECDC) in 2011/12 and the WHO in 2012/13 [14].

A comprehensive review of available literature pertaining to influenza in Montenegro has revealed an absence of studies in which one of the models for determining the threshold values for the epidemic period has been employed. The MEM method has never been used in Montenegro where surveillance of influenza-like illness (ILI) and acute respiratory infections (ARI) has been conducted since 2009 when WHO weekly reporting was also initiated.

The objective of this study was to evaluate the performance and usefulness of the MEM to establish whether it is appropriate method to be used as part of the influenza surveillance system in Montenegro.

## Method

Montenegro is located in south-east Europe, with the population of 620,045 inhabitants. Owing to its geographical position, there are several climate zones (Mediterranean, modified Mediterranean and temperate continental climate). The Influenza surveillance system in Montenegro is designed for monitoring ILI, ARI, laboratory-confirmed influenza cases and severe acute respiratory infection (SARI). ILI is defined as an acute respiratory illness with onset during the last 7 days with: measured temperature ≥ 38° and cough. ARI is defined as an acute onset of at least one of the following four respiratory symptoms: cough, sore throat, shortness of breath, coryza and a clinician’s judgment that the illness is due to an infection. ARI may present with or without fever [15].

Population surveillance of ILI and ARI is carried out throughout the calendar year and the weekly monitoring and reporting to WHO and ECDC is carried out during the influenza season (from calendar week 40–week 20 the following year). General practitioners and paediatricians in all primary healthcare centres in the country report ILI and ARI cases through electronic registration. This information is aggregated in the central database of the Institute of Public Health Montenegro [16].

Weekly data on the number of patients by age group with ILI and ARI, as well as the number of laboratory confirmed cases of influenza during the influenza season, were obtained from the Institute of Public Health of Montenegro.

## The Moving Epidemic Method (MEM)

The main purpose of the MEM is to calculate an epidemic threshold to serve as an alert signal for an upcoming epidemic. In addition, MEM calculates intensity thresholds to compare current epidemic intensity with previous epidemics identified from the same surveillance system as well as from other surveillance systems [14,17].

Using historical data (e.g. data from previous influenza seasons) from a specific surveillance system, the algorithm locates the timing of the influenza epidemic from each season (the MEM epidemic period) and separates it from pre-epidemic and post-epidemic activity. The epidemic threshold is calculated using the pre-epidemic values of historical seasons.

Intensity thresholds are calculated using the highest values of each epidemic period pooled together and calculating one-sided confidence intervals (CI) at several given levels. The three intensity threshold plus the epidemic threshold for five levels of intensity: baseline, low, medium, high and very high. In this paper, the geometric mean and levels of 40 (medium), 90 (high) and 97.5% (very high) has been used.

MEM gives estimations of the goodness of the method using a cross-validation procedure, comparing the weeks of each target season in the epidemic/non-epidemic periods (as isolated by the MEM algorithm) with weeks (of each target season) above/under the epidemic threshold calculated using the remaining seasons. In this context, sensitivity is defined as the number of epidemic weeks above the pre-epidemic threshold (before the peak) and above the post-epidemic threshold (after the peak) divided by the number of MEM epidemic weeks. Specificity pertains to the number of non-epidemic weeks below the pre-epidemic threshold (before the peak) and below the post-epidemic threshold (after the peak), divided by the number of MEM non-epidemic weeks. Positive predictive value (PPV) is obtained by dividing the number of epidemic weeks above the threshold by the number of weeks above the threshold, while negative predictive value (NPV) is calculated as the number of non-epidemic weeks below the threshold divided by the number of weeks below the threshold [18,19].

In this study, data from 2010/11–2017/18 influenza seasons were used. For a target season all the remaining seasons were used to calculate the epidemic threshold and the three intensity thresholds (medium, high and very high); summary statistics e.g. goodness statistics, peak value of the season, the week where the peak is reached and peak intensity level were also calculated.

For the 2017/18 influenza season, the number of false alerts and timeliness were also calculated. Here, false alert is defined as a weekly observed rate that is above the pre-epidemic threshold but is not in the MEM epidemic period. Timeliness pertains to the number of weeks between the alert week and the first week of the epidemic period as modelled by MEM. Statistical analysis was performed using the mem package of the R Language statistical software [20]. Graphs were produced using the memapp package, the MEM Wep Application using the Shiny Framework [21] and available from: www.memwebapp.com.

### Ethical statement

The Ethical Committee of the Faculty of Medicine, University of Belgrade, reviewed and approved the study (No 29/III-1; 28/3/2016).

## Results

### Modelled 2017/18 influenza season

**Figure 1:**
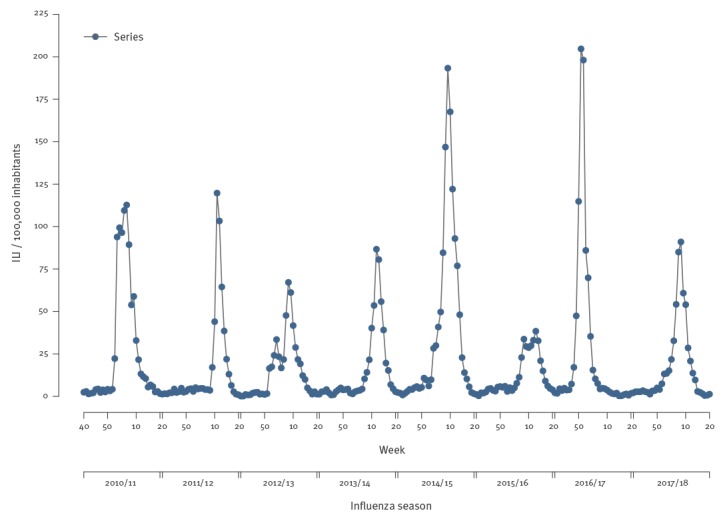
Time series of influenza-like illness, Montenegro, influenza seasons 2010/11–2017/18

The historical ILI time series used in this study is shown in Figure 1, indicating that the highest activity was recorded in 2016/17 and the lowest in the 2015/16 influenza season.

ILI: influenza-like illness.

**Figure 2:**
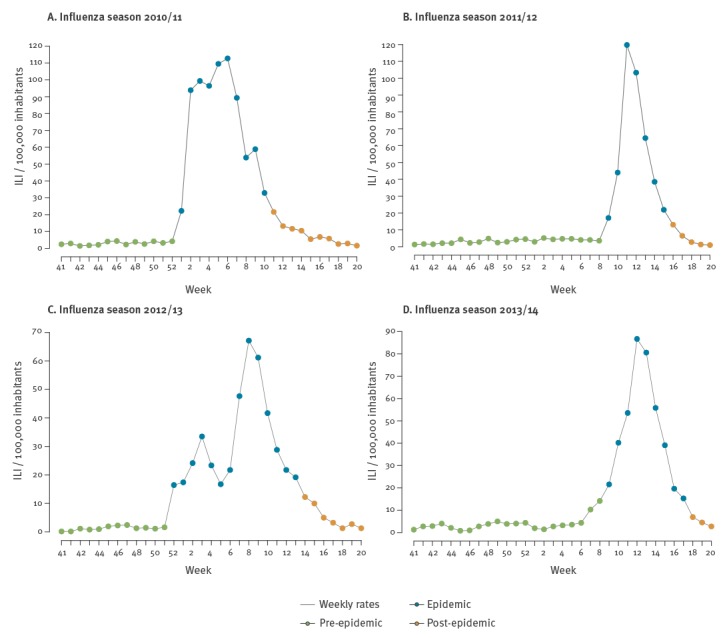
Flu wave periods, Montenegro, influenza seasons 2010/11–2013/14

**Figure 3:**
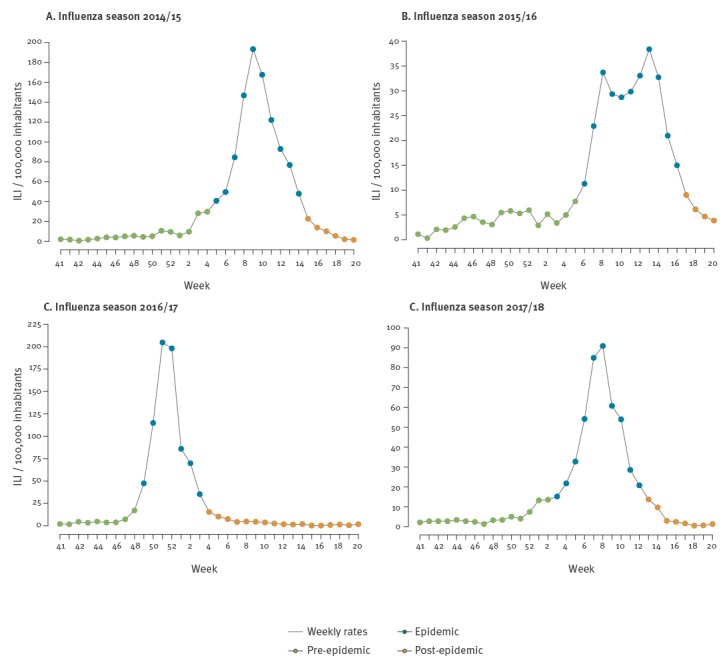
Flu wave periods, Montenegro, influenza seasons 2014/15–2017/18

**Figure 4:**
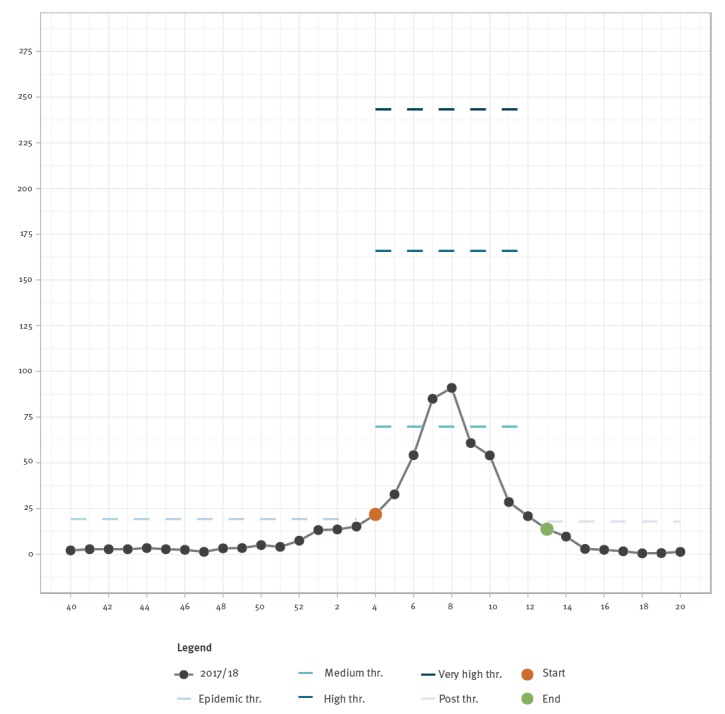
Moving Epidemic Method (MEM) epidemic threshold, levels of intensity and modelled influenza-like illness, Montenegro, influenza season 2017/18

The epidemic periods modelled by MEM for each season are presented in Figure 2 and 3. The pre-epidemic threshold per 100,000 inhabitants for ILI calculated for the 2017/18 target season was 19.23 while the post-epidemic threshold per 100,000 inhabitants was 17.55. In the observed 2017/18 season, by applying the MEM, an epidemic period of 10 weeks was identified. The fourth calendar week was identified as the alert week (the first week in the 2017/18 season with the disease incidence rate above the pre-epidemic threshold), as the MEM detected the epidemic onset (Figure 4). No false alerts were identified.

ILI: influenza-like illness.

ILI: influenza-like illness.

Thr: threshold.

The timeliness for the 2017/18 season in Montenegro was 0, the alert week and the first week of epidemic period (modelled by MEM) began at the same time. The intensity of the epidemic was medium, with the peak activity above the medium threshold (40% CI) of the historical epidemic level.

The cross validation procedure showed an excellent fit of the model (Table 1). The sensitivity of the epidemic threshold for the 2017/18 season was 89%, the specificity was 99% and the positive predictive value (PPV) and negative predictive value (NPV) were 97% and 96%, respectively. Epidemic and intensity thresholds, peaks and intensity levels from the 2010/11–2017/18 influenza seasons are presented in Table 2. The epidemic thresholds were the lowest (12.70) during influenza season 2014/15, ranging from 19.23 to 20.05 in the other influenza seasons. The peak intensity level was low in seasons 2012/13 and 2015/16, very high in 2014/15 and 2016/17 and medium in all the rest.

**Table 1:** Model goodness of fit, Montenegro, 2010/11–2017/18 influenza seasons

Influenza season	Sensitivity	Specificity	Positive predictive value	Negative predictive value
2010/11	1.00	0.94	0.87	1.00
2011/12	0.86	1.00	1.00	0.96
2012/13	0.81	1.00	1.00	0.88
2013/14	0.89	1.00	0.99	0.96
2014/15	1.00	0.87	0.78	1.00
2015/16	0.84	1.00	1.00	0.93
2016/17	0.95	1.00	1.00	0.99
2017/18	0.89	0.99	0.97	0.96
Total	0.90	0.98	0.94	0.96

**Table 2:** Epidemic and intensity thresholds, peaks and intensity levels, Montenegro, influenza seasons 2010/11–2016/17

Influenza season	Peak (ILI/100,000 inhabitants)	Peak week (ILI/100,000 inhabitants)	Epidemic threshold (ILI/100,000 inhabitants)	Medium threshold (ILI/100,000 inhabitants)	High threshold (ILI/100,000 inhabitants)	Very high threshold (ILI/100,000 inhabitants)	Peak level
2010/11	113	6	20.05	66.11	156.29	228.60	Medium
2011/12	120	11	20.05	69.20	161.36	234.60	Medium
2012/13	67	8	19.94	73.04	168.34	243.49	Low
2013/14	87	12	19.44	70.30	166.71	244.19	Medium
2014/15	193	9	12.70	63.47	136.07	190.61	Very high
2015/16	38	13	19.97	79.51	160.42	218.77	Low
2016/17	205	51	19.27	64.26	138.59	194.65	Very high
2017/18	91	8	19.23	69.77	165.91	243.30	Medium

ILI: influenza-like illness.

Thresholds were calculated based on data from all influenza seasons (2010/11–2017/18).

## Discussion

This study, is the first attempt to establish an epidemic threshold for the 2017/18 influenza season in Montenegro. To date, it has not been possible to determine the epidemic thresholds due to the lack of adequate historical data required for the analysis. The MEM was applied to data from eight influenza seasons, to calculate an epidemic threshold in Montenegro using ILI rates obtained through population surveillance for ILI during influenza seasons 2010/11–2016/17.

Based on the historical data, the pre-epidemic threshold for Montenegro for the observed 2017/18 season was ca 19 cases per 100,000 inhabitants. Lower values of pre-epidemic thresholds (based on the number of consultations in primary healthcare clinics per 100,000 inhabitants) for the 2017/18 season were registered in Wales (10.4) and England (13.1), while slightly higher values were recorded in Scotland (34.5), Northern Ireland (26.6) and Spain (55.7) [13,22]. The epidemic period lasted for 10 weeks, which is within the range (6–25 weeks) reported in other studies [8,23-26]. According to the available evidence, the duration of the influenza season in Europe ranges 12–19 weeks [27].

In Montenegro, the intensity of the influenza epidemic during the 2017/18 season was medium, with the peak in activity recorded above the ‘high’ threshold, based on the historical epidemic values. The high values of sensitivity, specificity, PPV, NPV indicate that the model fitted the data well and was able to predict epidemic thresholds with high certainty.

As MEM is an open method, it provides flexible procedures for calculating the threshold for non-hospitalised patients. The main advantage of MEM stems from the use of the algorithm that divides the influenza season into three periods using pre-epidemic information to determine the threshold, which was successfully implemented in this study. The high sensitivity and specificity of the threshold in detecting the onset of the epidemic was also shown, despite the differences between the data collection systems used in Montenegro compared with other countries in the region and the variations in data quality. These findings confirm the effectiveness of the model in meeting the needs of public health services.

In Montenegro, 95% of population is covered by influenza surveillance (although the electronic control system does not include private healthcare institutions, very few operate in the country). Although the MEM can also be applied to historical ARI data, in this study, we used exclusively ILI data for the eight seasons included. According to the studies conducted in other countries, MEM has shown better performance when applied to the ILI data, thus justifying our research strategy [14].

In extant research in this field, the number of seasons used for calculation of epidemic threshold using MEM ranged from five to 18 [14,17]. However, authors of several extant studies used fewer than five seasons to calculate duration of the influenza epidemic [13]. Unlike other methods, MEM does not take into account viral data, it is a model based solely on simple epidemiological data and represents the most practical choice for a standard approach to be adopted in the region and in Montenegro.

Determining the onset of an influenza epidemic is very important for many reasons. Each seasonal influenza epidemic presents an organisational challenge for healthcare systems. Timely information on the onset and the intensity of the influenza epidemic, is also important for the optimal deployment of human resources especially at regional level, as well as for the provision of sufficient quantities of medications [28,29]. Such a threshold can be a reminder to vaccinate members of society that are at risk of adverse influenza effects. The epidemic threshold, among other monitoring indicators, is used to make decisions about prescription of antiviral drugs, to facilitate identification of high-risk patients and increase accuracy of clinical diagnosis, as well as prompt taking samples for laboratory testing [12].

The ECDC advocates determining the epidemic threshold by applying the MEM. The reporting in Europe started in the 2011/12 season, whereas the Euro Flu Influenza Platform (WHO European Region) has been in use since 2012/13 [17]. Consequently, following its approval, the MEM and the resulting threshold have been in use in several countries for routine reporting about influenza season [22,23,30-32]. Epidemic thresholds are useful as a warning system, but should always be interpreted in conjunction with other available sources of information.

Our investigation was based on data pertaining to eight influenza seasons, which coincides with the period processed in the previously conducted surveys in 19/28 European Union countries [14]. Given the different conditions in Montenegro compared with those in other countries in the region, specifically differences in reporting and data collection methods as well as demographic characteristics and secular trends, the results from this study will contribute to the efforts to establish how best the model can fit available data, while also allowing the performance of various types of comparisons to be evaluated.

The establishment of one common method for analysing and interpreting the ILI data across Europe is the ultimate goal. The MEM could be a great option based on its intuitive concept, simple data requirements and flexibility compared with other sophisticated mathematical models. Influenza surveillance from Montenegro is included in the surveillance system already in place throughout Europe and therefore contributes to better quality of monitoring and surveillance of influenza epidemics in Europe.
